# MUC1-C activates EZH2 expression and function in human cancer cells

**DOI:** 10.1038/s41598-017-07850-0

**Published:** 2017-08-07

**Authors:** Hasan Rajabi, Masayuki Hiraki, Ashujit Tagde, Maroof Alam, Audrey Bouillez, Camilla L. Christensen, Mehmet Samur, Kwok-Kin Wong, Donald Kufe

**Affiliations:** Dana-Farber Cancer Institute Harvard Medical School Boston, Boston, MA 02215 USA

## Abstract

The EZH2 histone methyltransferase is a member of the polycomb repressive complex 2 (PRC2) that is highly expressed in diverse human cancers and is associated with a poor prognosis. MUC1-C is an oncoprotein that is similarly overexpressed in carcinomas and has been linked to epigenetic regulation. A role for MUC1-C in regulating EZH2 and histone methylation is not known. Here, we demonstrate that targeting MUC1-C in diverse human carcinoma cells downregulates EZH2 and other PRC2 components. MUC1-C activates (i) the *EZH2* promoter through induction of the pRB→E2F pathway, and (ii) an NF-κB p65 driven enhancer in exon 1. We also show that MUC1-C binds directly to the EZH2 CXC region adjacent to the catalytic SET domain and associates with EZH2 on the *CDH1* and *BRCA1* promoters. In concert with these results, targeting MUC1-C downregulates EZH2 function as evidenced by (i) global and promoter-specific decreases in H3K27 trimethylation (H3K27me3), and (ii) activation of tumor suppressor genes, including *BRCA1*. These findings highlight a previously unreported role for MUC1-C in activating EZH2 expression and function in cancer cells.

## Introduction

Histone methylation plays an essential role in the epigenetic control of gene expression in cancer^1, 2^. The polycomb group (PcG) proteins repress gene expression by maintaining chromatin in a transcriptionally suppressed state and thereby contribute to cell fate, development and cancer^1, 3, 4^. The PcG proteins form the (i) polycomb repressive complex 2 (PRC2), which predominantly catalyzes trimethylation of histone H3 at lysine 27 (H3K27me3), and (ii) polycomb repressive complex 1 (PRC1), which recognizes H3K27me3 and stabilizes the inactive epigenetic state^1, 4^. The PRC2 complex includes in part the enhancer of zeste homolog 2 (EZH2), suppressor of zeste 12 homolog (SUZ12) and embryonic ectoderm development (EED). EZH2 is a histone methyltransferase (HMT), which is dependent on the presence of SUZ12 and EED, and mediates H3K27 trimethylation with the downregulation of target genes^5, 6^. Overexpression of EZH2 in invasive and metastatic breast cancers is associated with a poor prognosis^7, 8^. EZH2 overexpression promotes tumorigenesis in mouse models of lung cancer^9^ and has been linked to poor clinical outcomes in patients with non-small cell lung cancer (NSCLC), as well as other types of carcinomas^10–16^. In concert with these findings, EZH2 confers a proliferative advantage, induces transformation and drives the epithelial-mesenchymal transition (EMT) program^11, 17–19^. The EZH2-containing PRC2 complex also recruits DNA methyltransferases (DNMTs) and thereby promotes the repression of tumor suppressor genes (TSGs), such as *CDH1*, by methylation of their promoters^2, 20–23^. Overexpression of EZH2 is associated with amplification of the *EZH2* locus in certain cancers^11^. In addition, activation of the E2F pathway contributes to *EZH2* transcription^11^. MYC has also been linked to activation of *EZH2* transcription and the regulation of EZH2 mRNA levels by a miR-26a-dependent mechanism^24–26^.

Mucin 1 (MUC1) is a heterodimeric protein that is aberrantly overexpressed in breast, non-small cell lung (NSCL) and other cancers^27^. Notably, MUC1 consists of two subunits^27^. The MUC1 N-terminal subunit (MUC1-N) is the mucin component of the heterodimer, which is positioned extracellularly in a complex with the transmembrane C-terminal subunit (MUC1-C)^27^. The MUC1-N/MUC1-C complex evolved to protect epithelia from stress by (i) a MUC1-N-associated physical barrier and (ii) a MUC1-C-activated signaling cascade that confers self-renewal, repair and survival^27, 28^. In this capacity and with overexpression in cancer, MUC1-C functions as an oncoprotein that interacts with (i) receptor tyrosine kinases (RTKs) at the cell surface and (ii) certain transcription factors, such as β-catenin/TCF4 and NF-κB p65, in the nucleus^29–31^. For example, MUC1-C activates the *MYC* gene by a β-catenin/TCF4-mediated mechanism^32, 33^. In turn, the MUC1-C→MYC pathway drives *BMI1* gene transcription and the ubiquitylation of H2A^34^. MUC1-C also activates the inflammatory TAK1→IKK→NF-κB pathway^29, 35–37^. The MUC1-C cytoplasmic domain binds directly to NF-κB p65 and promotes NF-κB p65 occupancy on the promoters of its target genes^29^. In this way, MUC1-C drives NF-κB-mediated activation of the *ZEB1* gene, suppresses miR-200c expression and promotes EMT^37^. The interaction between MUC1-C and NF-κB also promotes self-renewal capacity of carcinoma cells, activation of the LIN28B→let-7 pathway, downregulation of E-cadherin and expression of other markers of stemness^38, 39^. These findings and the demonstration that MUC1-C drives DNMT expression have supported the notion that MUC1-C links the inflammatory NF-κB pathway to epigenetic regulatory mechanisms associated with EMT and a malignant phenotype^40, 41^.

The present studies demonstrate that targeting MUC1-C in carcinoma cells is associated with downregulation of EZH2, SUZ12 and EED expression, indicating that MUC1-C activates major components of the PRC2 complex. We have focused here on MUC1-C-mediated regulation of EZH2 and demonstrate that MUC1-C drives *EZH2* transcription by retinoblastoma protein (pRB)→E2F- and NF-κB p65-mediated mechanisms. We further demonstrate that MUC1-C interacts directly with EZH2 and forms a complex with EZH2 on the *CDH1* and *BRCA1* promoters. In concert with these results, we show that targeting MUC1-C decreases global and gene promoter-specific H3K27me3 levels. These findings uncover a previously unrecognized role for MUC1-C in driving EZH2-mediated epigenetic regulation in cancer cells.

## Results

### MUC1-C drives EZH2 expression

**Figure 1:**
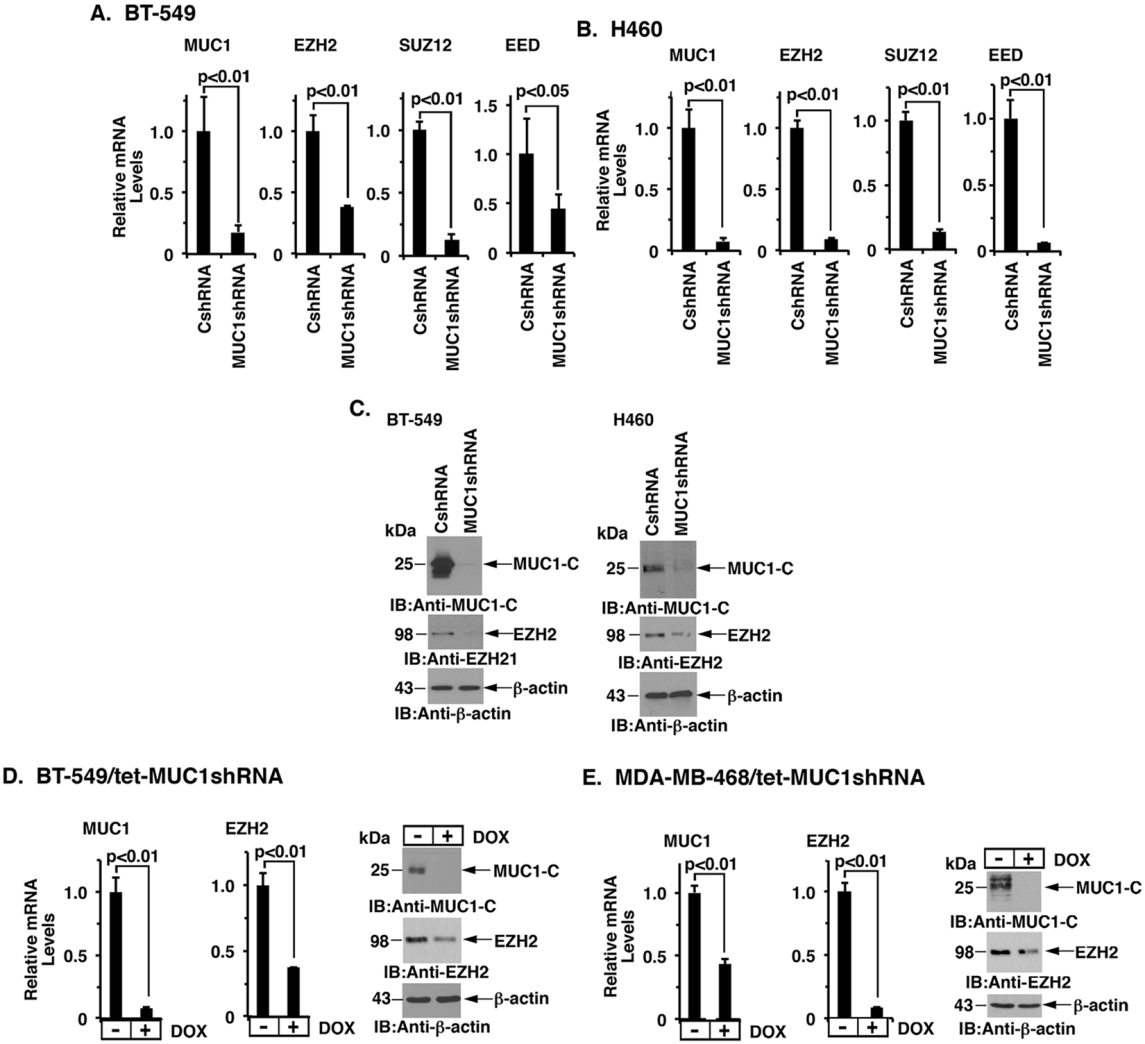
Silencing MUC1-C suppresses EZH2, SUZ12 and EED expression. A and B. BT-549 (**A**) and H460 (**B**) cells stably expressing a control scrambled shRNA (CshRNA) or a MUC1shRNA were analyzed for MUC1, EZH2, SUZ12 and EED mRNA levels by qRT-PCR using primers listed in Table S1. The results (mean ± SD) are expressed as relative mRNA levels compared to that obtained for the CshRNA cells (assigned a value of 1). (**C**) The respective BT-549 (left) and H460 (right) cells expressing a CshRNA or MUC1shRNA were immunoblotted with the indicated antibodies. (**D** and **E**) BT-549 (**D**) and MDA-MB-468 (**E**) cells were stably transduced to express a tetracycline-inducible MUC1 shRNA (tet-MUC1shRNA). Cells treated with 200 ng/ml DOX for 4 d were analyzed for MUC1 and EZH2 mRNA levels by qRT-PCR. The results (mean ± SD) are expressed as relative mRNA levels compared to that obtained for control DOX-untreated cells (assigned a value of 1) (left). Lysates from cells treated with 200 ng/ml DOX for 7 d were immunoblotted with the indicated antibodies (right). See also Fig. S1.

EZH2, a member of the PRC2 complex, has been linked to breast and NSCL cancers, among others. We found that stable silencing of MUC1-C in BT-549 triple-negative breast cancer (TNBC) cells is associated with downregulation of EZH2 mRNA levels (Fig. 1A). The PRC2 complex also includes SUZ12 and EED^1^ and, interestingly, silencing MUC1-C was associated with downregulation of SUZ12 and EED mRNA (Fig. 1A). Similar results were obtained in MDA-MB-231 (Supplemental Fig. S1A) and H460 (Fig. 1B) cells, indicating that MUC1-C drives EZH2, SUZ12 and EED expression in TNBC and NSCLC cells. EZH2 possesses HMT activity, whereas SUZ12 and EED are necessary for EZH2 function^42^. Accordingly, we focused our studies here on the regulation of EZH2. In concert with the mRNA results, targeting MUC1-C resulted in suppression of EZH2 protein (Fig. 1C, left and right). To extend these observations, we established BT-549 cells stably expressing a tetracycline-inducible MUC1 shRNA (tet-MUC1shRNA) or a control shRNA (tet-CshRNA). Treatment of BT-549/tet-MUC1shRNA cells with doxycycline (DOX) for 7 days resulted in suppression of MUC1-C and EZH2 expression (Fig. 1D, left and right). By contrast, treatment of BT-549/tet-CshRNA cells with DOX had no effect on MUC1-C or EZH2 mRNA levels (Supplemental Fig. S1B). Similar results were obtained with DOX-treated MDA-MB-468/tet-MUC1shRNA and MDA-MB-468/tet-CshRNA breast cancer cells (Fig. 1E, left and right; Supplemental Fig. S1C). We also found that silencing MUC1-C in KRAS mutant A549 NSCLC cells decreases EZH2 mRNA levels (Supplemental Fig. S1D). Moreover, MUC1-C was necessary for EZH2 expression in DU145 prostate cancer cells (Supplemental Fig. S1E), supporting the notion that MUC1-C drives the upregulation of EZH2 in diverse types of cancer cells.

### Targeting the MUC1-C cytoplasmic domain suppresses EZH2 expression

**Figure 2:**
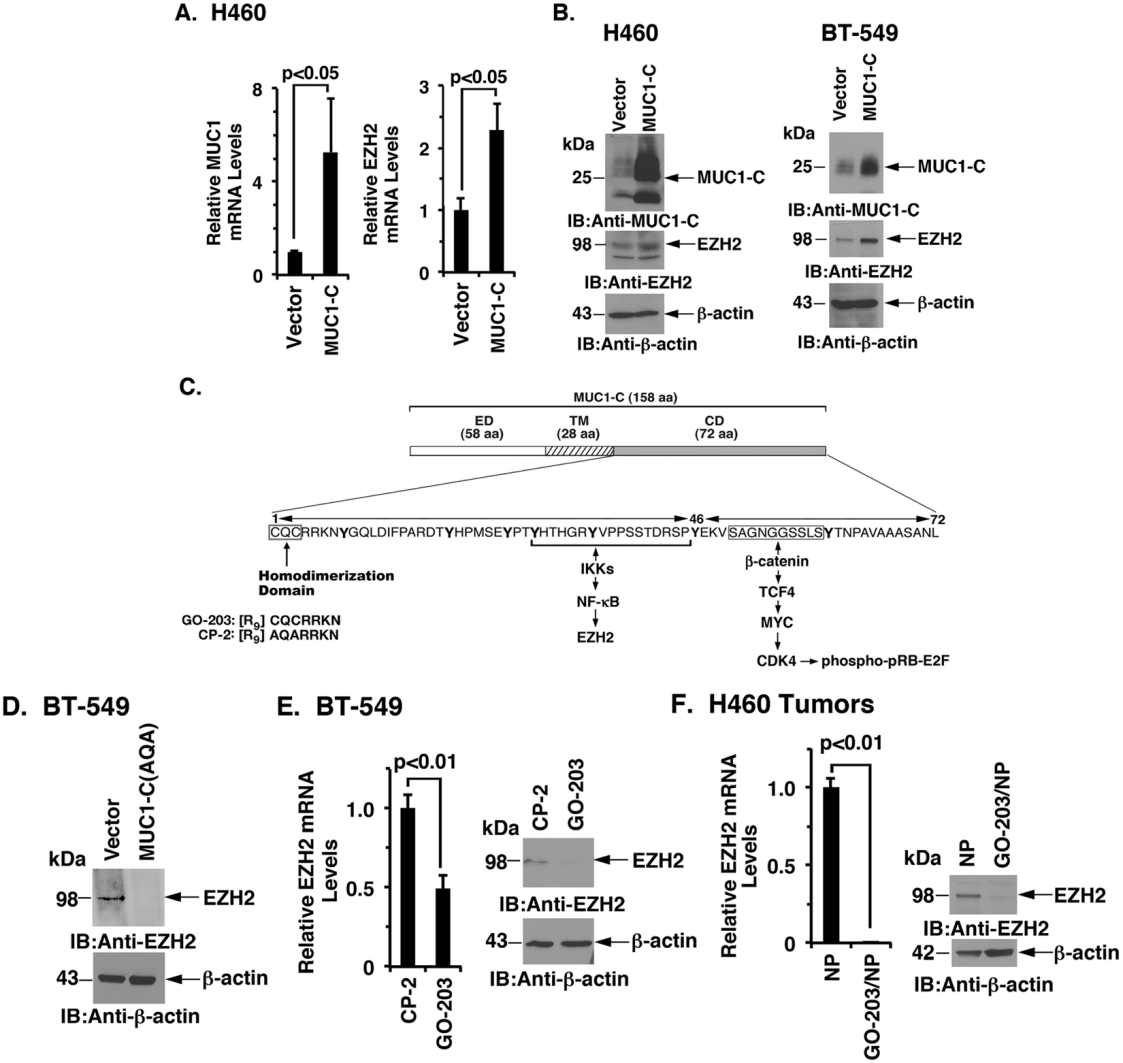
Targeting the MUC1-C cytoplasmic domain downregulates EZH2 expression. (**A**) H460 cells stably expressing a control or MUC1-C vector were analyzed for MUC1 (left) and EZH2 (right) mRNA levels by qRT-PCR. The results (mean ± SD) are expressed as relative mRNA levels compared to that obtained for vector cells (assigned a value of 1). (**B**) Lysates from H460 (left) and BT-549 (right) stably expressing a control or MUC1-C vector were immunoblotted with the indicated antibodies. (**C**) Schema of the MUC1-C subunit with the extracellular domain (ED), transmembrane domain (TM), and the sequence of the 72 aa cytoplasmic domain (CD). MUC1-CD contains a CQC motif that is necessary for MUC1-C homodimerization and oncogenic function. GO-203 is a cell-penetrating peptide that binds the CQC motif and blocks the formation of MUC1-C homodimers. Highlighted are MUC1-C-induced pathways that confer the activation of NF-κB p65 and MYC. (**D**) BT-549 cells were transfected with a control or MUC1-C(AQA) vector in which the CQC motif was mutated to AQA. Lysates were immunoblotted with the indicated antibodies. (**E**) BT-549 cells treated with 5 μM CP-2 or 5 μM GO-203 for 12 h were analyzed for EZH2 mRNA levels by qRT-PCR. The results (mean ± SD) are expressed as relative EZH2 mRNA levels compared to that obtained for CP-2 (assigned a value of 1) (left). Lysates from cells treated with 5 μM CP-2 or 5 μM GO-203 for 48 h were immunoblotted with the indicated antibodies (right). (**F**) H460 tumors obtained on day 14 of treatment with empty NPs or GO-203/NPs^32^ were analyzed for EZH2 mRNA levels by qRT-PCR. The results (mean ± SD) are expressed as relative EZH2 mRNA levels compared to that obtained for empty NP-treated cells (assigned a value of 1) (left). Lysates were immunoblotted with the indicated antibodies (right).

In concert with the above findings, enforced overexpression of MUC1-C resulted in upregulation of EZH2 mRNA and protein (Fig. 2A,B), demonstrating that MUC1-C, and not the MUC1 N-terminal subunit (MUC1-N), is necessary for this response. The MUC1-C subunit consists of a 72-amino acid (aa) intrinsically disordered cytoplasmic domain that is sufficient for promoting self-renewal and tumorigenicity (Fig. 2C)^30, 43^. Noteworthy is the presence of a CQC motif in the MUC1-C cytoplasmic domain that is required for the formation of MUC1-C homodimers and for MUC1-C-mediated transformation (Fig. 2C)^44, 45^. Moreover, expression of MUC1-C in which the CQC motif is mutated to AQA suppresses tumorigenicity, consistent with a dominant-negative effect for transformation^44, 45^. In support of a role for MUC1-C in driving EZH2, expression of the MUC1-C(AQA) mutant resulted in downregulation of EZH2 levels (Fig. 2D). The MUC1-C inhibitor GO-203 (Fig. 2C) binds to the MUC1-C CQC motif and blocks MUC1-C homodimerization and its oncogenic function^46, 47^. Treatment of BT-549 cells with GO-203, but not with the control peptide CP-2, was associated with downregulation of EZH2 expression (Fig. 2E, left and right). GO-203 has been formulated in polymeric nanoparticles (NPs) for delivery to tumors in mouse models^48^. Treatment of H460 tumor xenografts with GO-203/NPs^32^ resulted in marked downregulation of EZH2 (Fig. 2F, left and right), supporting the premise that targeting the MUC1-C cytoplasmic domain *in vitro* and *in vivo* is sufficient for suppression of EZH2 expression.

### MUC1-C activates *EZH2* transcription by an E2F-dependent mechanism

**Figure 3:**
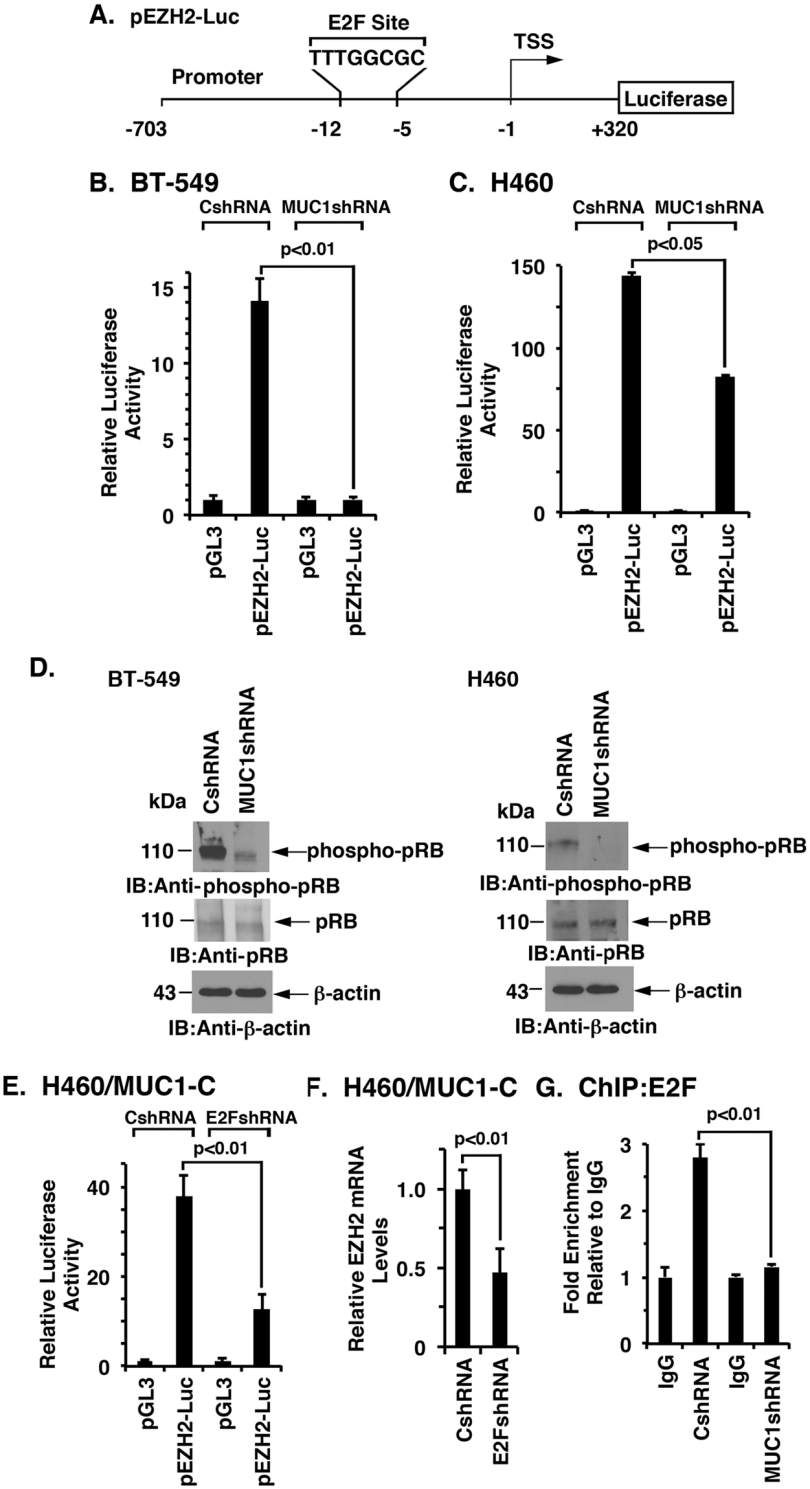
Targeting MUC1-C suppresses the EZH2 promoter. (**A**) Schema of the pEZH2-Luc vector with positioning of the E2F binding site at −12 to −5 bp upstream of the transcription start site. (**B** and **C**) The respective BT-549 (**B**) and H460 (**C**) cells expressing a CshRNA or MUC1shRNA were transfected with the pGL3-Basic Luc or pEZH2-Luc reporter for 48 h and then analyzed for luciferase activity. The results (mean ± SD of 3 determinations) are expressed as the relative luciferase activity compared to that obtained with pGL3-Basic Luc (assigned a value of 1). (**D**) Lysates from the respective BT-549 (left) and H460 (right) cells expressing a CshRNA or MUC1shRNA were immunoblotted with the indicated antibodies. (**E**) H460/MUC1-C cells were transduced with lentiviral vectors to express a control shRNA (CshRNA) or a E2F shRNA. The cells were transfected with the pGL3-Basic Luc or pEZH2-Luc reporter for 48 h and then analyzed for luciferase activity. The results (mean ± SD of 3 determinations) are expressed as the relative luciferase activity compared to that obtained with pGL3-Basic Luc (assigned a value of 1). (**F**) The respective H460/MUC1-C cells were analyzed for EZH2 mRNA levels by qRT-PCR. The results (mean ± SD) are expressed as relative mRNA levels compared to that obtained for the CshRNA cells (assigned a value of 1). (**G**) Soluble chromatin from H460/CshRNA and H460/MUC1shRNA cells was precipitated with anti-E2F or a control IgG. The final DNA samples were amplified by qPCR with primers for the *EZH2* promoter. The results (mean ± SD of three determinations) are expressed as the relative fold enrichment compared with that for the control IgG (assigned a value of 1).

Studies with a pEZH2-luciferase promoter-reporter (pEZH2-Luc) (Fig. 3A) demonstrated that targeting MUC1-C in BT-549 cells is associated with a decrease in promoter activity (Fig. 3B). Silencing MUC1-C in H460 cells also decreased pEZH2-Luc activation (Fig. 3C), indicating that MUC1-C drives *EZH2* transcription. The *EZH2* promoter includes a consensus E2F binding site (TTTGGCGC) (Fig. 3A)^11, 49, 50^; however, there is no known association between MUC1-C and E2F-mediated gene transcription. Nonetheless, MUC1-C has been linked to the induction of CDK4 and phosphorylation of pRB (phospho-pRB)(Fig. 2C, schema)^32^. In this respect, we found that silencing MUC1-C decreases phospho-pRB levels (Fig. 3D, left and right) and, accordingly, we asked if MUC1-C induces *EZH2* transcription by a pRB→E2F-mediated mechanism. Indeed, silencing E2F in H460/MUC1-C cells was associated with downregulation of pEZH2-Luc activity (Fig. 3E) and EZH2 expression (Fig. 3F). Moreover, ChIP studies demonstrated that silencing MUC1-C decreases occupancy of E2F on the *EZH2* promoter (Fig. 3G), supporting the notion that MUC1-C→E2F signaling activates the *EZH2* promoter.

### MUC1-C enhances *EZH2* activation by an NF-κB-mediated mechanism

**Figure 4:**
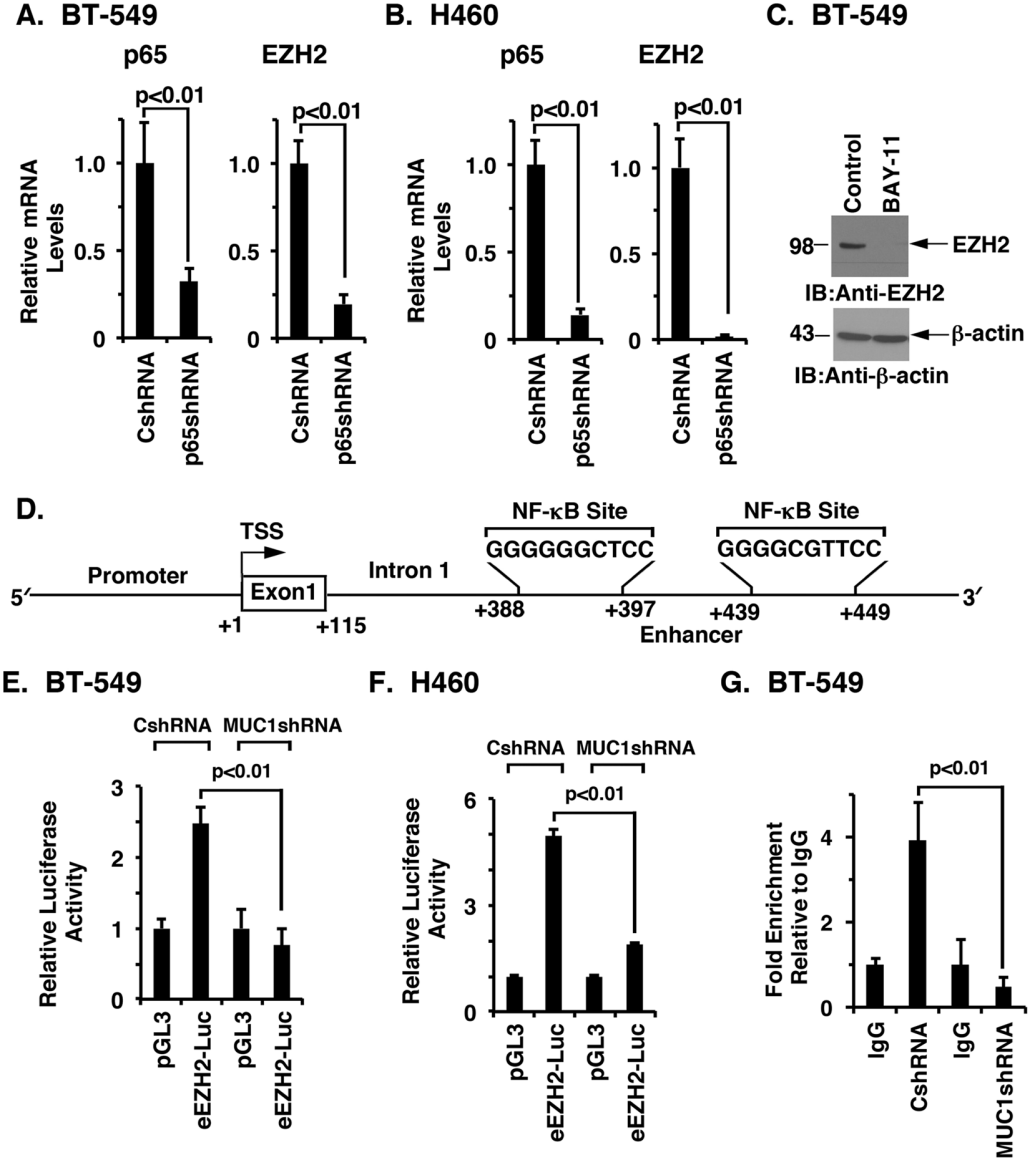
MUC1-C activates an enhancer in the EZH2 intron 1 by an NF-κB-medicated mechanism. A and B. BT-549 (**A**) and H460 (**B**) cells were transduced with lentiviral vectors to stably express a control shRNA (CshRNA) or a NF-κB p65 shRNA. The indicated cells were analyzed for NF-κB p65 (left) and EZH2 (right) mRNA levels by qRT-PCR using primers listed in Table S1. The results (mean ± SD) are expressed as relative EZH2 mRNA levels compared to that obtained for CshRNA cells (assigned a value of 1). (**C**) Lysates from BT-549 cells treated with 5 μM BAY-11-7085 or vehicle control for 48 h were immunoblotted with the indicated antibodies. (**D**) Schema of the *EZH2* intron 1 region with positioning of the putative NF-κB binding sites downstream of the transcription start site. (**E** and **F**) The respective BT-549 (**E**) and H460 (**F**) cells expressing a CshRNA or MUC1shRNA were transfected with the pGL3-Basic Luc or eEZH2-Luc reporter for 48 h and then analyzed for luciferase activity. The results (mean ± SD of 3 determinations) are expressed as the relative luciferase activity compared to that obtained with pGL3-Basic Luc (assigned a value of 1). (**G**) Soluble chromatin from BT-549/CshRNA and BT-549/MUC1shRNA cells was precipitated with anti-NF-κB p65 or a control IgG. The final DNA samples were amplified by qPCR with primers for the *EZH2* intron 1 region (Table S2). The results (mean ± SD of three determinations) are expressed as the relative fold enrichment compared with that obtained with the IgG control (assigned a value of 1).

MUC1-C activates the inflammatory TAK1→IKK→NF-κB pathway^36^ and thereby induces *DNMT1* and *DNMT3b* expression^40^. Based on these findings and the known integration of EZH2 with DNMTs and DNA methylation^2, 20–23^, we asked if MUC1-C also regulates EZH2 expression by an NF-κB-mediated mechanism. Intriguingly, we found that (i) silencing NF-κB p65 decreases EZH2 mRNA (Figs 4A,B, left and right), and (ii) treatment with the NF-κB inhibitor BAY-11-7085 decreases EZH2 protein (Fig. 4C). We therefore searched for putative NF-κB binding sites and identified two consensus sequences downstream to the transcription start site at positions + 388 to + 397 and + 439 to + 449 in the first *EZH2* intron (Fig. 4D). In support of a potential enhancer function, incorporation of the intron 1 fragment containing two putative NF-κB binding sites in a luciferase reporter (eEZH2-Luc) demonstrated activation in BT-549 and H460 cells by a MUC1-C-dependent mechanism (Fig. 4E,F). Additionally, we found that NF-κB p65 occupies the *EZH2* intron 1 region and that silencing MUC1-C suppresses NF-κB occupancy (Fig. 4G). These and the above findings indicate that MUC1-C (i) activates the *EZH2* promoter by E2F-dependent signaling, and (ii) enhances *EZH2* transcription by an NF-κB p65-mediated mechanism.

### MUC1-C binds directly to EZH2

**Figure 5:**
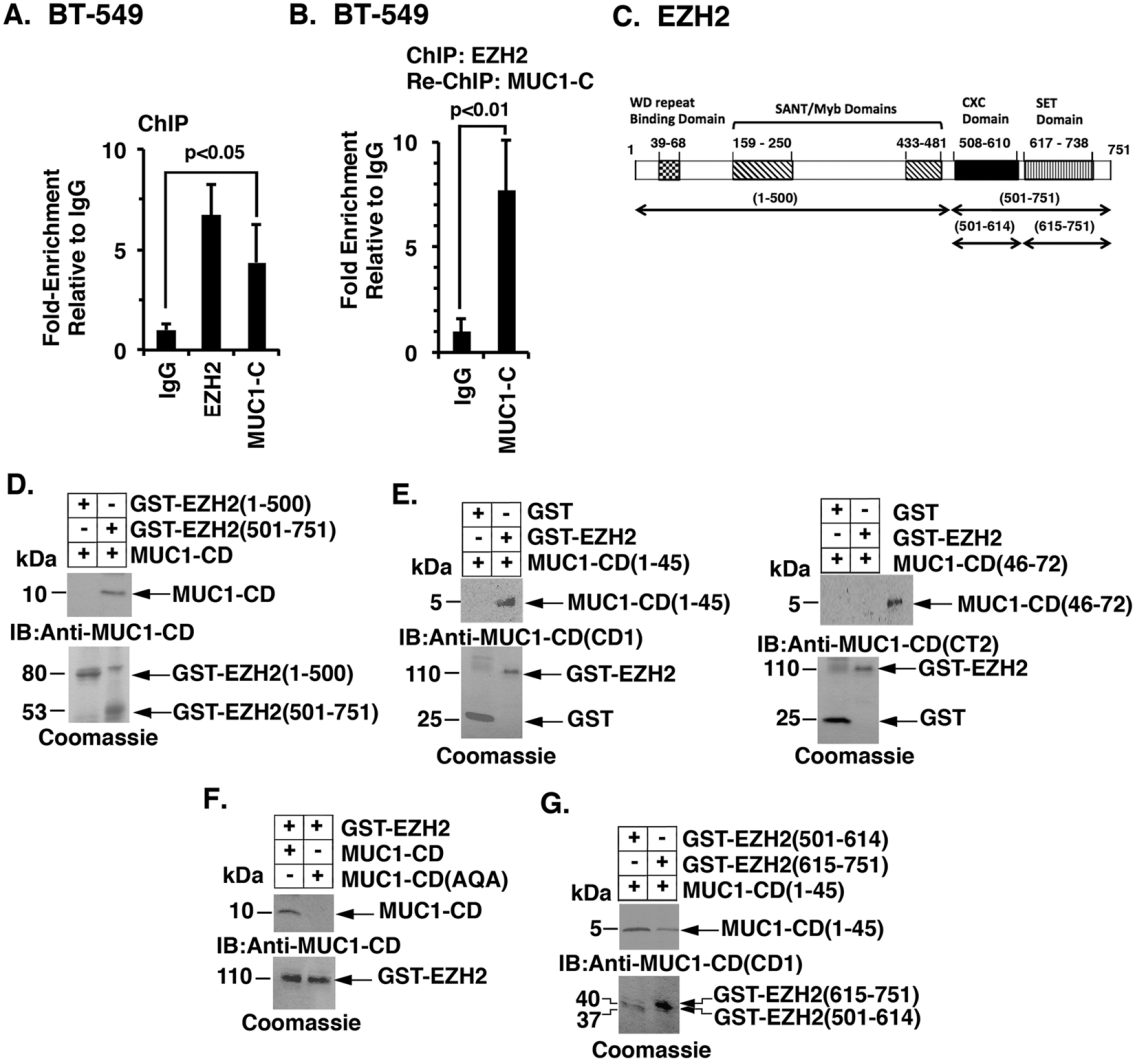
MUC1-C forms complexes with EZH2 by binding directly to the EZH2 CXC domain. (**A**) Soluble chromatin from BT-549 cells was precipitated with anti-EZH2, anti-MUC1-C or a control IgG. The final DNA samples were amplified by qPCR with primers for the *CDH1* promoter (Table S2). The results (mean ± SD of three determinations) are expressed as the relative fold enrichment compared with that obtained with the IgG control (assigned a value of 1). (**B**) In the re-ChIP analysis, anti-EZH2 precipitates were released and re-immunoprecipitated with anti-MUC1-C or a control IgG. The final DNA samples were amplified by qPCR with primers for the *CDH1* promoter. The results (mean ± SD of three determinations) are expressed as the relative fold enrichment compared with that obtained with the IgG control (assigned a value of 1). See also Fig. S2. (**C**) Schema of the 751 aa EZH2 protein highlighting the WD-repeat binding domain, two SANT/Myb domains, a CXC domain and a SET domain that catalyzes methylation of H3K27. Also highlighted are the GST-EZH2 fragments used for direct binding studies. (**D**) GST-EZH2(1-500) and GST-EZH2(501–751) were incubated with purified MUC1-C cytoplasmic domain (MUC1-CD). The adsorbates were immunoblotted with anti-MUC1-CD. Input of the GST proteins was assessed by Coomassie blue staining. (**E**) GST and GST-EZH2 were incubated with purified MUC1-CD(1–45) (left) or MUC1-CD(46–72) (right). The adsorbates were immunoblotted with the appropriate anti-MUC1-CD antibody (CD1, left; CT2, right). Input of the GST proteins was assessed by Coomassie blue staining. (**F**) GST and GST-EZH2 were incubated with purified MUC1-CD and MUC1-CD(AQA). The adsorbates were immunoblotted with anti-MUC1-CD. Input of the GST proteins was assessed by Coomassie blue staining. (**G**) GST-EZH2(501–614) and GST-EZH2(615–751) were incubated with purified MUC1-CD(1–45). The adsorbates were immunoblotted with anti-MUC1-CD (MAb CD1). Input of the GST proteins was assessed by Coomassie blue staining

MUC1-C interacts with certain transcriptional complexes^28^ and contributes to the recruitment of epigenetic regulators, such as the histone acetyltransferase p300^31, 32^. To determine if MUC1-C interacts with EZH2, we performed ChIP studies on the *CDH1* promoter, which is a target for EZH2-mediated repression^18, 19^ and found occupancy of both EZH2 and MUC1-C (Fig. 5A). Re-ChIP studies further showed that EZH2 and MUC1-C form a complex on the *CDH1* promoter (Fig. 5B). Similar results were obtained in studies of the *CDH1* promoter in H460 cells; that is, (i) occupancy by both MUC1-C and EZH2 (Supplemental Fig. S2A), and (ii) detection of MUC1-C/EZH2 complexes (Supplemental Fig. S2B). EZH2 consists of 751 aa, which include a WD-repeat binding domain, two adjacent SANT/Myb domains, a CXC domain and a SET domain that catalyzes methylation of H3K27 (NCBI Accession NM_004456; Fig. 5C)^51^. To further assess the nature of the association between EZH2 and MUC1-C, we first generated GST-EZH2 fragments that included aa 1–500 and 501–751 (Fig. 5C). Incubation of these fragments with the MUC1-C cytoplasmic domain (MUC1-CD) demonstrated binding to EZH2(501–751), and not EZH2(1–500) (Fig. 5D), supporting a direct interaction. Based on these results, we incubated GST-EZH2 with MUC1-CD fragments and found that MUC1-CD(1–45), and not MUC1-CD(46–72), confers the interaction (Fig. 5E, left and right). MUC1-CD contains a CQC motif at residues 1–3 that is necessary for interactions with certain binding partners (Fig. 2C)^31, 47^. Mutation of both Cys residues to Ala (AQA) blocked the interaction between MUC1-CD and EZH2 (Fig. 5F). The EZH2(501–751) fragment includes a CXC domain (aa 508 to 610) and the SET catalytic domain (aa 617 to 738). Accordingly, we generated GST-EZH2(501–614) and GST-EZH2(615–751). Incubation of these fragments with MUC1-CD(1–45) demonstrated substantially higher binding with EZH2(501–614) as compared with EZH2(615–751) (Fig. 5G). These findings demonstrate that the MUC1-C cytoplasmic domain interacts predominantly with EZH2 at the CXC region adjacent to the catalytic SET domain.

### Targeting MUC1-C decreases global and CDH1 promoter-specific H3K27 trimethylation

**Figure 6:**
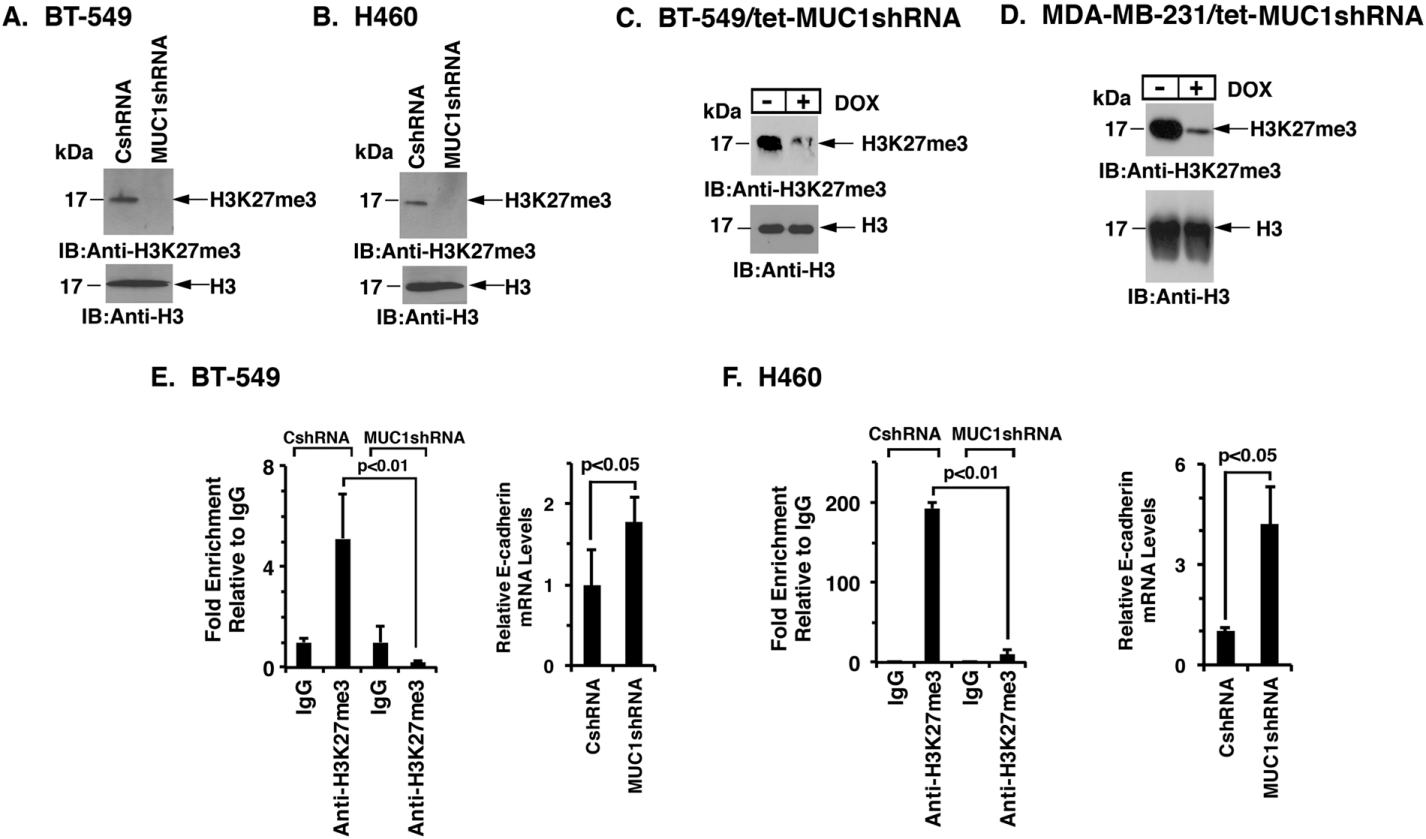
Targeting MUC1-C represses global and CDH1 promoter-specific H3K27me3 levels. (**A** and **B**) Lysates from the respective BT-549 (**A**) and H460 (**B**) cells expressing a CshRNA or MUC1shRNA were immunoblotted with anti-H3K27me3 and anti-histone H3. (**C** and **D**) Lysates from BT-549/tet-MUC1shRNA (**C**) and MDA-MB-231/tetMUC1shRNA (**D**) cells treated with 200 ng/ml DOX for 4 d were immunoblotted with the indicated antibodies. E and F. Soluble chromatin from the respective BT-549 (**E**) and H460 (**F**) cells expressing a CshRNA or MUC1shRNA was precipitated with anti-H3K27me3 or a control IgG. The final DNA samples were amplified by qPCR with primers for the *CDH1* promoter. The results (mean ± SD of three determinations) are expressed as the relative fold enrichment compared with that obtained with the IgG control (assigned a value of 1) (left). Cells were also analyzed for E-cadherin mRNA levels by qRT-PCR using primers listed in Table S1. The results (mean ± SD) are expressed as relative mRNA levels compared to that obtained for the CshRNA cells (assigned a value of 1) (right). See also Fig. S3.

The demonstration that MUC1-C induces EZH2 expression and binds directly to EZH2 prompted studies to assess the effects of targeting MUC1-C on global H3K27 trimethylation. We found that silencing MUC1-C in BT-549 cells is associated with decreases in global H3K27me3 levels (Fig. 6A). Similar results were obtained in H460 cells (Fig. 6B). Treatment of BT-549/tet-MUC1shRNA (Fig. 6C) and MDA-MB-231/tet-MUC1shRNA (Fig. 6D) cells with DOX was also associated with downregulation of H3K27me3. In addition, overexpression of MUC1-C increased H3K27me3 levels (Supplemental Fig. S3A). In concert with the findings that MUC1-C/NF-κB p65 complexes activate *EZH2* transcription, we also found that targeting NF-κB p65 with silencing or BAY-11-7085 decreases H3K27me3 levels (Supplemental Fig. S3B–D). ChIP studies on the *CDH1* promoter further demonstrated that silencing MUC1-C decreases H3K27me3 levels in association with increases in E-cadherin expression (Fig. 6E,F, left and right), providing further support for the notion that MUC1-C drives EZH2-mediated H3K27 trimethylation.

### MUC1-C→EZH2 signaling represses expression of the BRCA1 tumor suppressor

**Figure 7:**
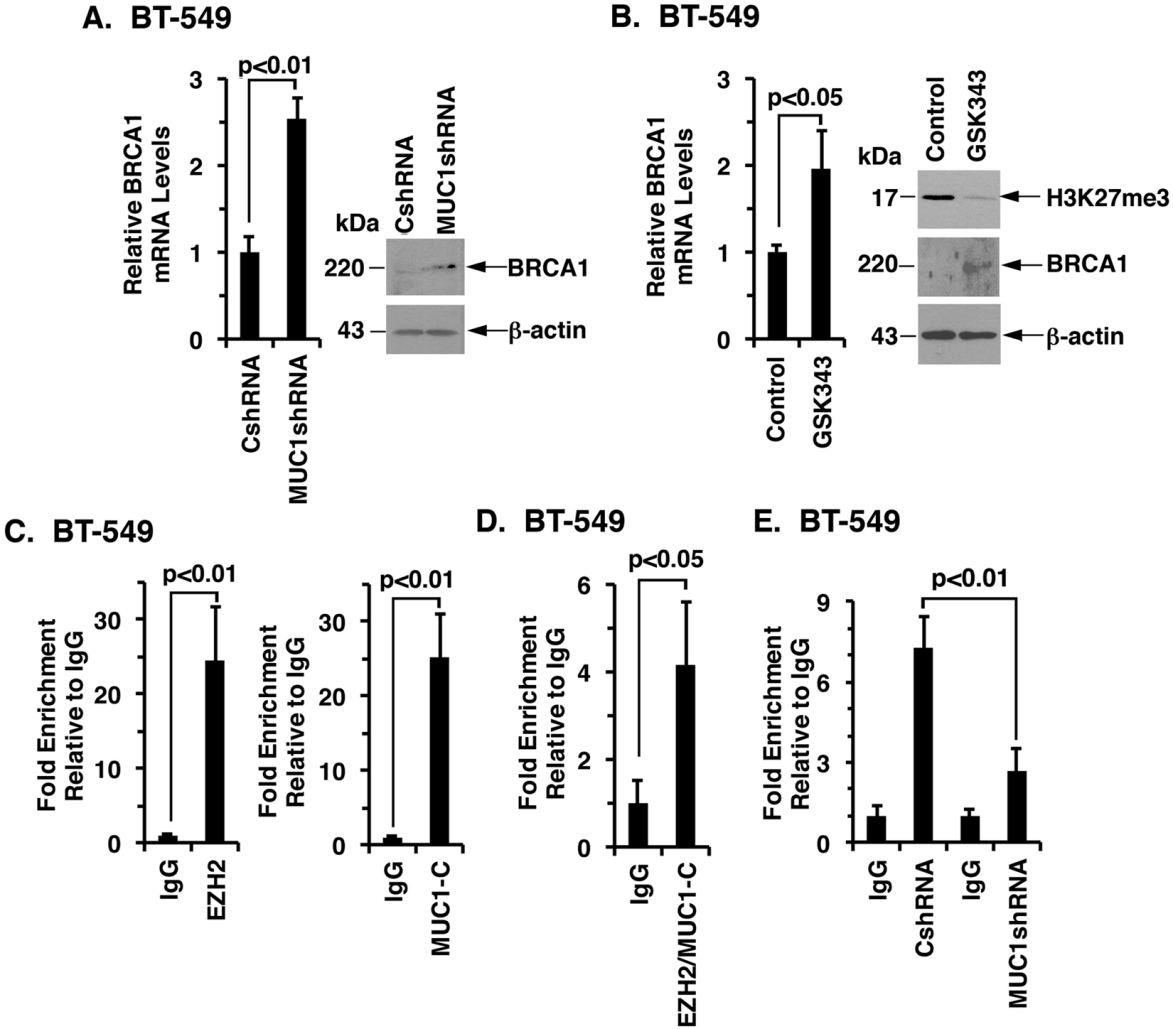
Targeting MUC1-C activates the BRCA1 promoter by suppressing EZH2 occupancy and H3K27me3 levels. (**A**) BT-549/CshRNA and BT-549/MUC1shRNA cells were analyzed for BRCA1 mRNA levels by qRT-PCR. The results (mean ± SD) are expressed as relative mRNA levels compared to that obtained for the CshRNA cells (assigned a value of 1) (left). Lysates were immunoblotted with the indicated antibodies (right). (**B**) BT-549 cells treated with vehicle control or 10 μM GSK343 for 72 h were analyzed for BRCA1 mRNA levels by qRT-PCR. The results (mean ± SD) are expressed as relative mRNA levels compared to that obtained for the Control cells (assigned a value of 1) (left). Lysates were immunoblotted with the indicated antibodies (right). (**C**) Soluble chromatin from BT-549 cells was precipitated with anti-EZH2 (left), anti-MUC1-C (right) or a control IgG. (**D**) In the re-ChIP analysis, EZH2 precipitates were released and re-immunoprecipitated with anti-MUC1-C and a control IgG. (**E**) Soluble chromatin from BT-549/CshRNA and BT-549/MUC1shRNA cells was precipitated with anti-H3K27me3 or a control IgG. The final DNA samples were amplified by qPCR with primers for the *BRCA1* promoter. The results (mean ± SD of three determinations) are expressed as the relative fold enrichment compared with that obtained with the IgG control (assigned a value of 1).

To extend this investigation of the MUC1-C→EZH2 pathway, we performed RNA-seq analysis of cells without and with MUC1 silencing. An unanticipated outcome was the finding of a highly significant (p < 1 × 10^−12^) relationship with up- and down-regulated genes encoding effectors of the DNA damage response, including *BRCA1*, *CHK2* and *RAD51*, among many others (Supplemental Fig. S4A). In keeping with the focus of the present work, we confirmed that MUC1 expression negatively correlates with that of BRCA1 in datasets from breast cancers (Supplemental Fig. S4B) and NSCLCs (Supplemental Fig. S4C). In addition, silencing MUC1 was associated with upregulation of BRCA1 in BT-549 (Fig. 7A, left and right) and H460 (Supplemental Fig. S5A, left and right) cells. MUC1-C has been linked to the repression of TSGs by DNMT- and PRC1-mediated epigenetic mechanisms^34, 40^. However, to our knowledge there is no reported association between MUC1 or EZH2 and *BRCA1* gene repression. We therefore treated cells with the EZH2 inhibitor GSK343 and found upregulation of BRCA1 mRNA and protein levels (Fig. 7B, left and right; Supplemental Fig. S5B, left and right), indicating that, like MUC1-C, targeting EZH2 induces BRCA1 expression. ChIP studies further demonstrated that both MUC1-C and EZH2 occupy the *BRCA1* promoter (Fig. 7C, left and right; Supplemental Fig. S5C, left and right). Re-ChIP experiments also showed that MUC1-C and EZH2 form a complex on the *BRCA1* promoter (Fig. 7D; Supplemental Fig. S5D). Moreover, silencing MUC1-C was associated with suppression of H3K27 trimethylation of the *BRCA1* promoter (Fig. 7E; Supplemental Fig. S5E), supporting a model in which the MUC1-C→EZH2→H3K27me3 pathway promotes repression of the *BRCA1* gene.

## Discussion

**Figure 8:**
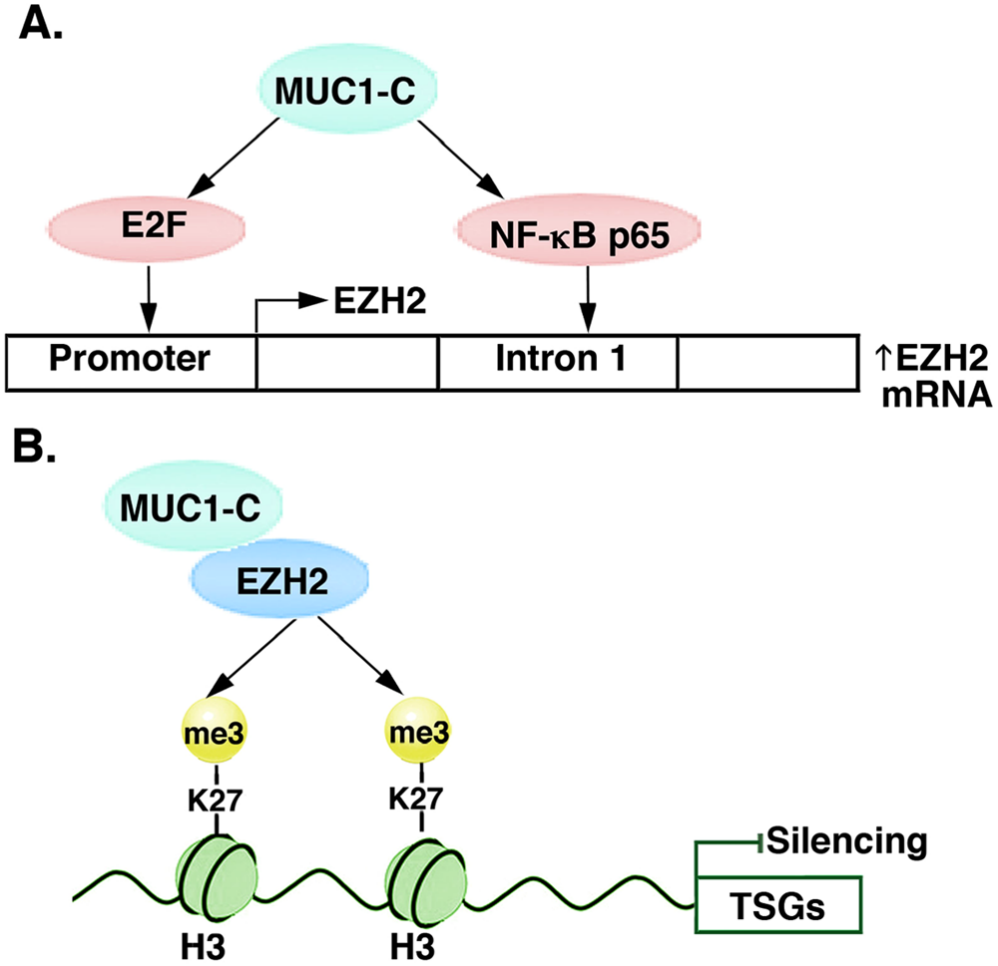
Schemas depicting the proposed MUC1-C-induced regulation of EZH2 expression and function in epigenetic repression. (**A**) MUC1-C drives EZH2 expression by inducing (i) the pRB→E2F pathway and in turn E2F-mediated activation of the *EZH2* promoter, and (ii) NF-κB p65 occupancy of the *EZH2* intron 1 and enhancing *EZH2* transcription. (**B**) MUC1-C binds directly to EZH2, increases EZH2 occupancy on TSG promoters and enhances EZH2-mediated H3K27 trimethylation with repression of gene expression.

EZH2 has emerged as a highly attractive target based on its elevated expression in human carcinomas and association with poor clinical outcomes^52^. Gain- and loss-of-function mutations in EZH2 have also been identified in certain hematologic malignancies^53–55^. In addition, CML stem cells are dependent on EZH2 for survival^56, 57^, further supporting the need for agents that target EZH2 and the PRC2 complex. Indeed, EZH2 has been proposed as a master regulator of gene transcription in the promotion of cancer^6, 52^. The present studies demonstrate that MUC1-C drives *EZH2* expression in TNBC, NSCLC and other types of carcinoma cells. Additionally, we found that MUC1-C promotes the expression of SUZ12 and EED. Therefore, targeting MUC1-C can inactivate the PRC2 complex in multiple ways, including downregulation of EZH2, as well as suppression of SUZ12 and EED, which are required for EZH2 HMT activity^1^. We focused here on how MUC1-C activates *EZH2* based largely on its dysregulation in cancer. Accordingly, subsequent work will be needed to address the role of MUC1-C in driving SUZ12 and EED expression. MUC1-C induces *MYC* transcription by activation of the β-catenin/TCF4 pathway^32, 33^. Thus, targeting MUC1-C decreases expression of MYC and its downstream target genes, such as *CDK4* ^32^. In turn, targeting MUC1-C indirectly suppresses pRB activity^32^. The present results uncover a previously unrecognized role for MUC1-C in activation of the pRB→E2F pathway and thereby the *EZH2* promoter (Fig. 8). Interestingly, pRB→E2F signaling has also been shown to activate *EED* gene transcription^11^. Indeed, in the course of these experiments, we found that MUC1-C also activates *EED* expression by a pRB→E2F-mediated mechanism.

The MUC1-C cytoplasmic domain activates the β-catenin/TCF4 pathway by binding directly to β-catenin and promoting β-catenin occupancy on promoters of WNT target genes, such as *CCND1* and *MYC* ^30–33^. The MUC1-C cytoplasmic domain also promotes activation of the TAK1→IKK→NF-κB inflammatory pathway, binds directly to NF-κB p65 and promotes occupancy of NF-κB p65 on its target genes, including *ZEB1* and *LIN28B*, among others^29, 35–37, 39^. Overexpression of MUC1-C in carcinomas thereby subverts the NF-κB pathway in driving the induction of EMT^37, 39^. The effects of MUC1-C on NF-κB p65 activation have also been linked to induction of self-renewal capacity and stemness of cancer cells^38, 39^. Such characteristics of EMT, self-renewal and stemness depend, at least in part, on epigenetic regulatory mechanisms involving PRC2 to achieve the associated changes in gene expression patterns^2^. However, to our knowledge, there had been no known link between MUC1-C→NF-κB signaling and the induction of *EZH2* expression. In searching for such evidence, we found that MUC1-C/NF-κB p65 complexes occupy consensus NF-κB binding sites in the *EZH2* first intron and activate *EZH2* transcription. These results and those obtained with E2F support a model in which MUC1-C induces *EZH2* expression by the β-catenin/TCF4→MYC and the NF-κB pathways (Fig. 8). Of note, our findings do not exclude the possibility that MUC1-C regulates EZH2 expression by additional mechanisms. For instance, MYC suppresses miR-26a, which targets EZH2 mRNA^25, 26, 58^. In addition, MUC1-C activates LIN28B and thereby suppresses let-7, another miRNA that targets EZH2 expression^39, 59^.

MUC1-C induces the expression of *DNMT1* and *DNMT3b*, but not *DNMT3a*, in carcinoma cells^40^. As a result, MUC1-C controls global and TSG promoter-specific DNA methylation^40^. Interestingly in this regard, EZH2 functions as a recruitment platform for DNMTs, linking H3K27 methylation and DNA methylation in gene repression^20, 21, 23^. An unexpected finding was that, in addition to inducing EZH2 expression in cancer cells, MUC1-C was detectable in complexes with EZH2 on the *CDH1* and *BRCA1* promoters, invoking the notion that MUC1-C associates with EZH2. EZH2 contains a WD repeat domain that is necessary for binding to EED and thereby activation of the catalytic HMT SET domain. EZH2 also includes SANT DNA binding domains and a highly conserved CXC domain that may contribute to an inactive configuration of the SET domain^60^. Our results demonstrate that the MUC1-C cytoplasmic domain CQC motif binds directly with the EZH2 CXC domain. The MUC1-C CQC motif is necessary and sufficient for the formation of MUC1-C homodimers and their import into the nucleus^44^. The MUC1-C CQC motif has also been shown to confer interactions with certain transcription factors, including TCF4 and others^31, 37, 61^, supporting the premise that this motif is also of importance for binding to nuclear proteins. MUC1-C may thus play dual roles in regulating EZH2; namely, (i) induction of EZH2 expression and (ii) direct binding to the EZH2 CXC motif and thereby affecting the SET domain HMT activity. In this regard, our results further demonstrate that MUC1-C forms a complex with EZH2 on the *CDH1* and *BRCA1* promoters and enhances H3K27 trimethylation of those regions (Fig. 8).

EZH2-mediated H3K27 trimethylation acts as a site for recruitment of (i) the PRC1 complex, and (ii) DNMTs, and thereby links these epigenetic mechanisms of gene silencing^1, 20^. MUC1-C is necessary for expression of PRC1 complex members, B cell-specific Moloney murine leukemia virus integration site 1 (BMI1), RING1 and RING2^34^. MUC1-C also binds directly to BMI1 and promotes occupancy of BMI1 on target promoters^34^. Given the diversity by which MUC1-C drives the functions of PRC2, PRC1 and DNMTs in epigenetic gene silencing, we performed RNA-seq on cells without and with MUC1-C silencing. The findings demonstrated that MUC1-C regulates diverse genes involved in DNA repair pathways. For instance, in the homologous recombination DNA repair pathway, we found that, like *BRCA1*, MUC1-C represses *CHK2* and *RAD51* expression by an EZH2-mediated mechanism (unpublished data). Targeting MUC1-C also activates genes in the mismatch repair, base-excision repair and DNA interstrand cross-link repair pathways, suggesting that the overexpression of MUC1-C as found in human carcinomas could contribute to genomic instability. One task at hand is to now investigate which of the potential MUC1-C-induced epigenetic changes involving PRC2, PRC1 and/or DNA methylation contribute to the downregulation of these additional DNA repair genes. The present findings and the involvement of MUC1-C in driving EMT and immune evasion thereby support the integration of multiple phenotypic characteristics of the cancer stem-like cell (CSC) and a mechanistic basis for the development of anti-cancer drug resistance^62^. Another task at hand is to target MUC1-C and thereby suppress this integrated CSC program in human tumors. For that purpose, the MUC1-C inhibitor, GO-203, has been evaluated in Phase I clinical trials and, based on a favorable safety profile, has been formulated in polymeric nanoparticles for sustained delivery to patients with MUC1-C-expressing cancers^48^.

## Experimental Procedures

### Cell culture

Human BT-549 breast cancer, H460 NSCLC, A549 NSCLC and DU145 prostate cancer cells were grown in RPMI1640 medium (ATCC, Manassas, VA, USA). MDA-MB-231 and MDA-MB-468 breast cells were cultured in Dulbecco’s modified Eagle’s medium (DMEM) (Corning, Manassas, VA, USA). BT-20 cells were cultured in Eagle’s Minimum Essential Medium (EMEM) (ATCC). Media were supplemented with 10% heat-inactivated fetal bovine serum (HI-FBS), 100 U/ml penicillin and 100 μg/ml streptomycin. Cells were transduced to stably express a control scrambled CshRNA or a MUC1 shRNA^40^. Cells stably expressing an empty vector or MUC1-C were generated as described^63^. Cells were treated with the MUC1-C inhibitor GO-203 or the control CP-2 peptide^38^. Cells were also treated with the NF-κB inhibitor BAY-11-7085 (Santa Cruz Biotechnology, Dallas, TX, USA) and the EZH2 inhibitor GSK343 (SelleckChem, Houston, TX, USA). Authentication of cells was performed by short tandem repeat (STR) analysis. Cell were monitored for mycoplasma contamination using the MycoAlert® Mycoplasma Detection Kit (Lonza, Rockland, ME, USA).

### Tetracycline-inducible MUC1 silencing

MUC1shRNA (MISSION shRNA; Sigma, TRCN0000122938) or a control scrambled CshRNA (Sigma) was inserted into the pLKO-tet-puro vector (Addgene, Cambridge, MA, USA; Plasmid #21915). The viral vectors were produced in HEK293T cells as previously described^63, 64^. BT-549 and MDA-MB-468 cells expressing tet-CshRNA or tet-MUC1shRNA were selected for growth in 1–3 μg/ml puromycin. Cells were treated with doxycycline (DOX; Sigma).

### Real-time quantitative reverse-transcription PCR (qRT-PCR)

Total RNA was isolated using with Trizol reagent (Invitrogen, Carlsbad, CA, USA). Complementary DNA was synthesized from 2.0 μg total RNA using the High Capacity cDNA Reverse Transcription Kit (Applied Biosystems, Grand Island, NY, USA)^65^. The Power SYBR Green PCR Master Mix (Applied Biosystems) was used with 1 μl of diluted cDNA for each sample. The samples were amplified using the 7300 Realtime PCR System (Applied Biosystems). Primers used for qRT–PCR analysis are listed in Supplemental Table S1.

### *EZH2* promoter and enhancer luciferase reporter assays

Cells growing in 24-well plates were transfected with (i) an empty pGL3 vector, (ii) a pEZH2-Luc containing EZH2 promoter sequences −703 to + 320 relative to the TSS (Active Motif, Carlsbad, CA, USA), or (ii) eEZH2-Luc containing EZH2 intron 1 sequences +115 to +615 bp downstream to the TSS, and SV-40-*Renilla*-Luc in the presence of Lipofectamine^TM^ 3000 Reagent (Invitrogen). At 48 h after transfection, cell extracts were prepared with passive lysis buffer using the Luciferase® Assay System (Promega, Madison, WI, USA). Luminescence was measured with the Dual-Luciferase® Reporter Assay System (Promega).

### Chromatin immunoprecipitation (ChIP) assay

Soluble chromatin was precipitated with anti-MUC1-C (NeoMarkers, Fremont, CA, USA), anti-E2F (Cell Signaling Technology, Danvers, MA, USA), anti-NF-κB p65 (Santa Cruz Biotechnology), anti-H3K27me3 (Abcam, Cambridge, MA, USA), anti-H3K27 (Abcam), or a control non-immune IgG (Santa Cruz Biotechnology). For re-ChIP analysis, complexes from the primary ChIP were eluted and re-immunoprecipitated with a secondary antibody. For real-time ChIP qPCR, the SYBR green system was used with the ABI Prism 7300 sequence detector (Applied Biosystems). Data are reported as relative-fold enrichment^40^. Primers used for ChIP qPCR are listed in the Supplementary Table S2.

### Immunoblot analysis

Whole cells were lysed in NP-40 buffer, containing protease inhibitor cocktail (ThermoScientific, Waltham, MA, USA). Immunoblotting was performed with anti-MUC1-C (NeoMarkers), anti-EZH2 (Cell Signaling Technology), anti-phospho-pRB, anti-pRB (BD Biosciences, San Jose, CA, USA), anti-H3K27me3 (Abcam), anti-E-cadherin (Cell Signaling Technology), anti-NF-κB p65 (Santa Cruz Biotechnology) and anti-β-actin (Sigma).

### Protein binding assays

GST-EZH2 was purified from pGEX-EZH2 (Addgene; Plasmid #28060). GST-EZH2(1–500), GST-EZH2(501–751), GST-EZH2(501–614) and GST-EZH2(615–751) fragments were generated by PCR amplification of the pGEX-EZH2 plasmid and cloning into the pGEX-5X-1 bacterial expression plasmid backbone (GE Healthcare, Piscataway, NJ, USA). MUC1-CD, MUC1-CD(AQA) and the MUC1-CD(1–45) and MUC1-CD(46–72) fragments were prepared by expressing the relevant GST-fusion proteins and cleaving the GST tag with thrombin as described^37^. GST and GST fusion proteins bound to glutathione beads were incubated with purified proteins, washed and the adsorbates were analyzed by immunoblotting with anti-MUC1-C cytoplasmic domain antibodies CD1^66^ and CT2 (NeoMarker).

### Statistical analysis

Each experiment was repeated at least three times. Data are expressed as mean ± SD. The unpaired Student’s t-test was used to examine differences between means of two groups. A p-value < 0.05 was considered a statistically significant difference.

## Electronic supplementary material

Supplementary Material
