# Supplementary material for: MUC1-C activates EZH2 expression and function in human cancer cells
Source: Sci Rep. 2017 Aug 7;7:7481. doi: 10.1038/s41598-017-07850-0 (PMC5547076; doi:10.1038/s41598-017-07850-0)

MUC1-C ACTIVATES EZH2 EXPRESSION AND FUNCTION IN HUMAN CANCER CELLS

Hasan Rajabi, Masayuki Hiraki, Ashujit Tagde, Maroof Alam, Audrey Bouillez, Camilla L. Christensen, Mehmet Samur, Kwok-Kin Wong and Donald Kufe*

Dana-Farber Cancer Institute
Harvard Medical School
Boston, MA 02215

***Corresponding Authors:** Donald Kufe, 450 Brookline Avenue, DA830, Boston, Massachusetts, 02215, 617-632-3141 Tel., 617-632-2934 Fax, donald_kufe@dfci.harvard.edu

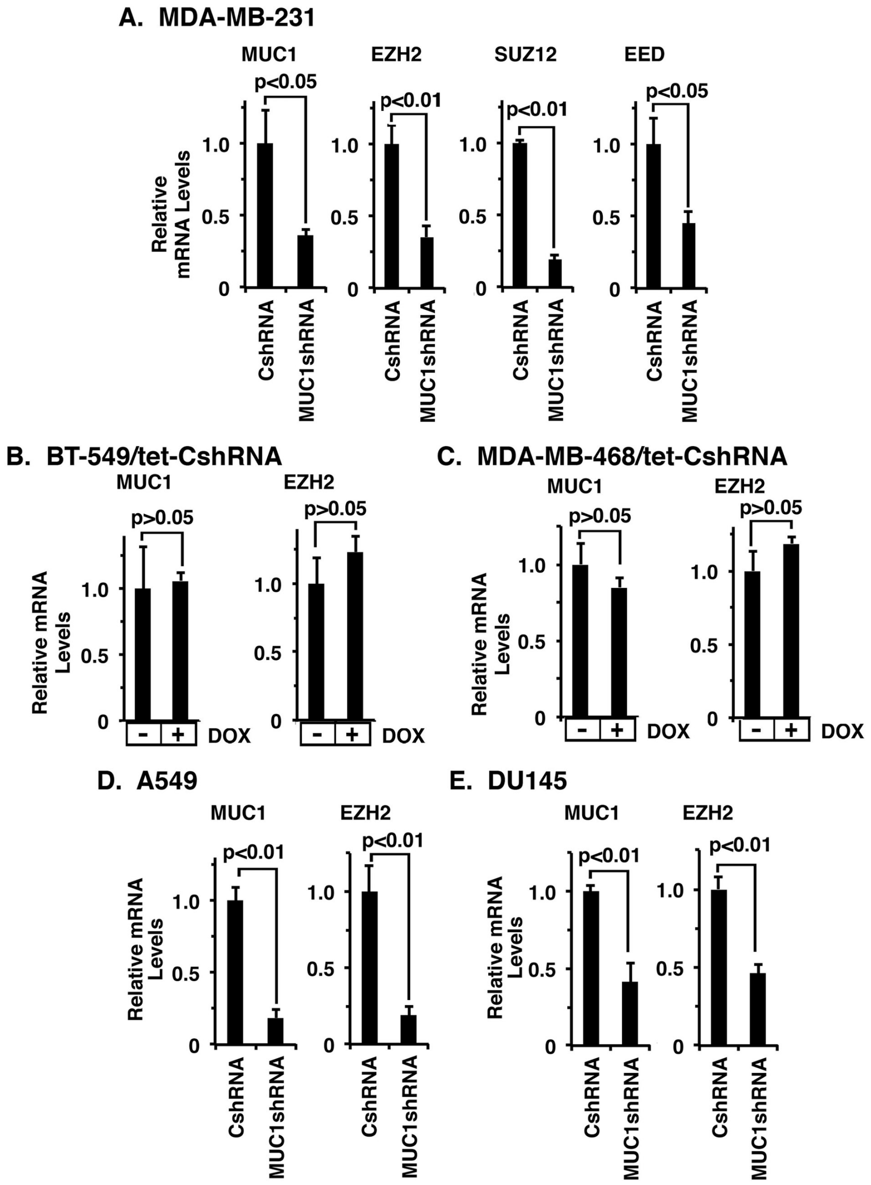

Figure S1. MUC1-C regulates EZH2 expression in diverse types of carcinoma cells. Relates to Figure 1. A. MDA-MB-231 cells stably expressing a control scrambled shRNA (CshRNA) or a MUC1shRNA were analyzed for MUC1, EZH2, SUZ12 and EED mRNA levels by qRT-PCR. The results (mean±SD) are expressed as relative mRNA levels compared to that obtained for the CshRNA cells (assigned a value of 1). B and C. BT-549 (B) and MDA-MB-468 (C) cells were stably transduced to express a tetracycline-inducible control scrambled shRNA (tet-CshRNA). Cells treated with 200 ng/ml DOX for 4 d were analyzed for MUC1 and EZH2 mRNA levels by qRT-PCR. The results (mean±SD) are expressed as relative mRNA levels compared to that obtained for control DOX-untreated cells (assigned a value of 1). D and E. A549 (D) and DU145 (E) cells stably expressing a control scrambled shRNA (CshRNA) or a MUC1shRNA were analyzed for MUC1 and EZH2 mRNA levels by qRT-PCR. The results (mean±SD) are expressed as relative mRNA levels compared to that obtained for the CshRNA cells (assigned a value of 1).

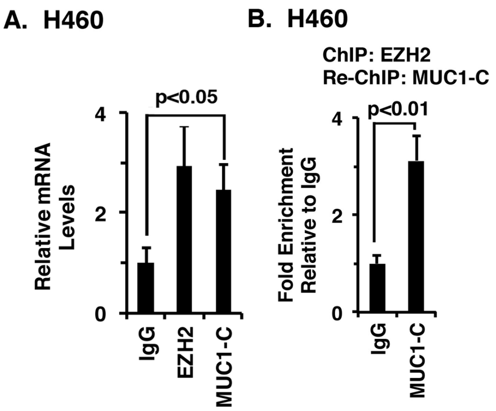

Figure S2. MUC1-C occupies the *CDH1* promoter in a complex with EZH2. Relates to Figure 6. A. Soluble chromatin from H460 cells was precipitated with anti-EZH2, anti-MUC1-C or a control IgG. The final DNA samples were amplified by qPCR with primers for the *CDH1* promoter (Table S2). The results (mean±SD of three determinations) are expressed as the relative fold enrichment compared with that obtained with the IgG control (assigned a value of 1). B. In the re-ChIP analysis, anti-EZH2 precipitates were released and re-immunoprecipitated with anti-MUC1-C or a control IgG. The final DNA samples were amplified by qPCR with primers for the *CDH1* promoter. The results (mean±SD of three determinations) are expressed as the relative fold enrichment compared with that obtained with the IgG control (assigned a value of 1).

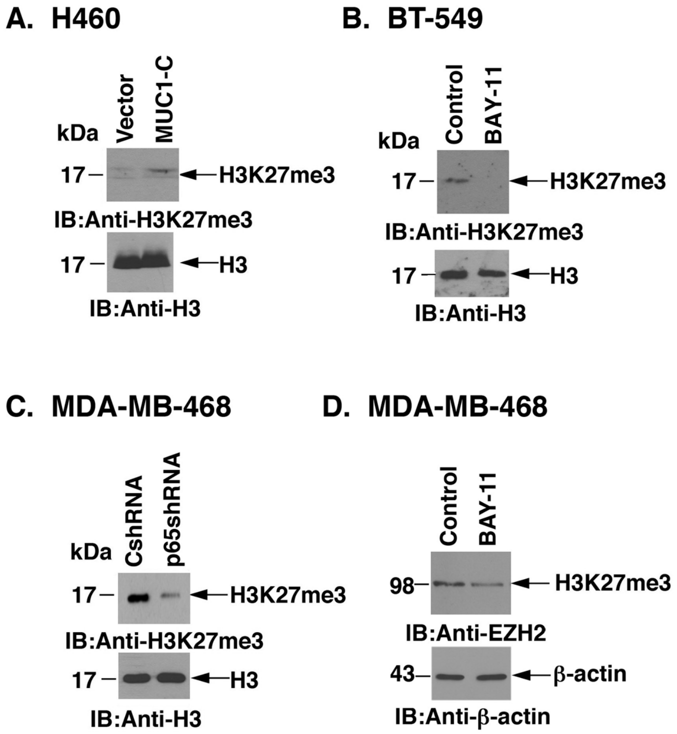

Figure S3. MUC1-C and NF-κB p65 drive H3K27 trimethylation. Relates to Figure 6. A. Lysates from H460/vector and H460/MUC1-C cells were immunoblotted with the indicated antibodies. B. Lysates from BT-549 cells treated with 5 μM BAY-11-7085 or vehicle control for 48 h were immunoblotted with the indicated antibodies. C. Lysates from the MDA-MB-468/vector and MDA-MB-468/p65shRNA cells were immunoblotted with the indicated antibodies. D. Lysates from MDA-MB-468 cells treated with 5 μM BAY-11-7085 or vehicle control for 48 h were immunoblotted with the indicated antibodies.

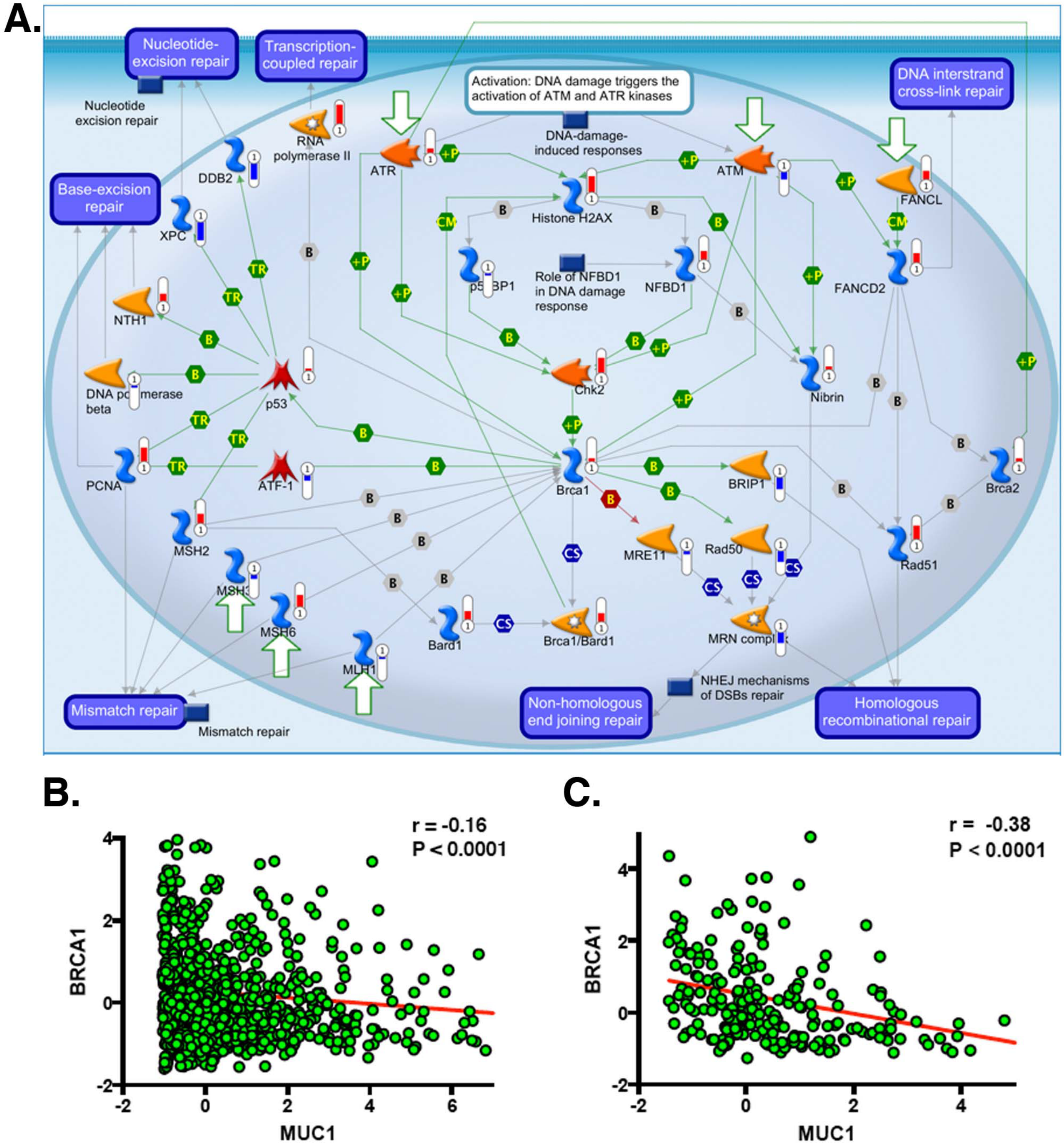

Figure S4. MUC1 expression correlates negatively with that of BRCA1. A. RNA-seq data from H460/CshRNA and H460/MUC1shRNA cells was analyzed using MetaCore for enrichment. B and C. *MUC1* and *BRCA1* gene expression data from TCGA datasets (cBioPortal) of breast (B; n=817) and lung (C; n=230) was assessed using the Spearman's correlation coefficient, where $p<0.05$ was considered as statistically significant.

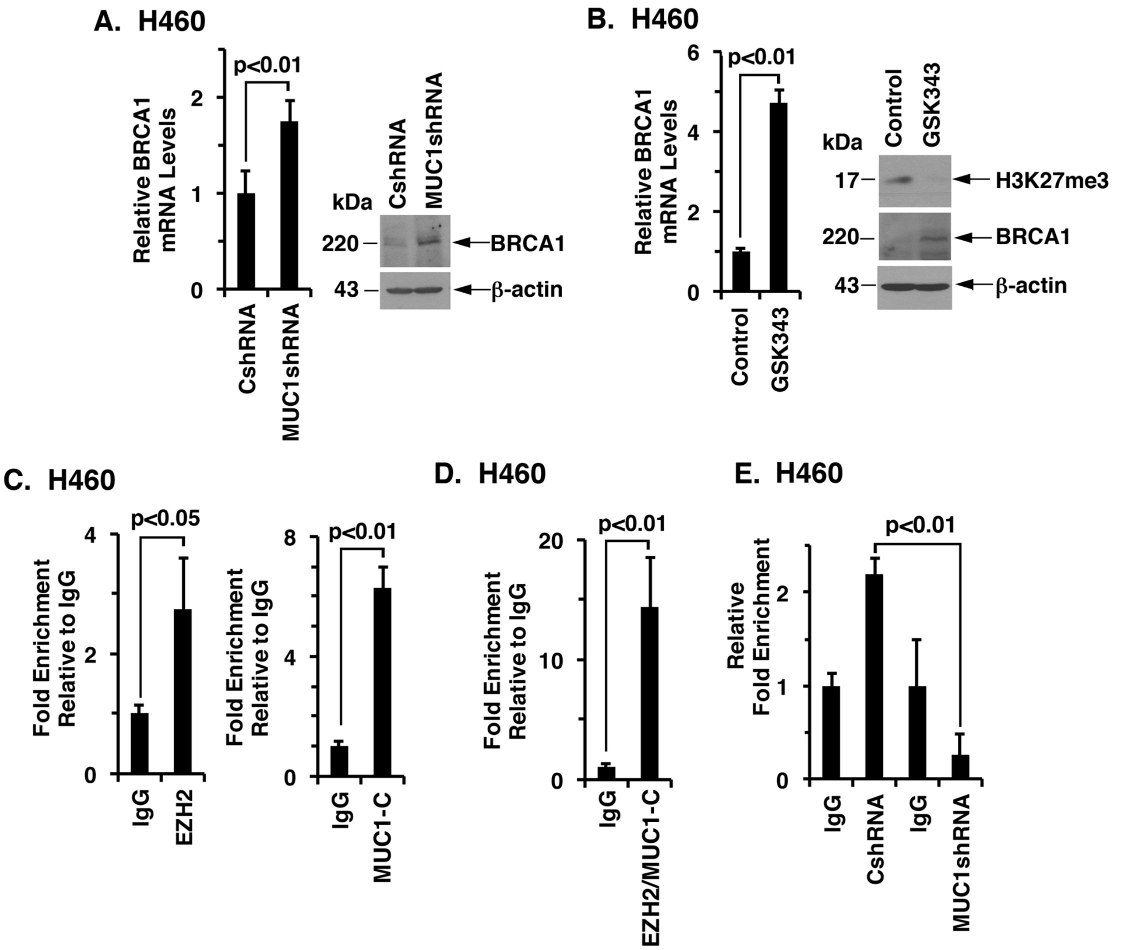

Figure S5. Targeting MUC1-C and EZH2 induces BRCA1 expression in H460 cells. A. H460/CshRNA and H460/MUC1shRNA cells were analyzed for BRCA1 mRNA levels by qRT-PCR. The results (mean±SD) are expressed as relative mRNA levels compared to that obtained for the CshRNA cells (assigned a value of 1)(left). Lysates were immunoblotted with the indicated antibodies (right). B. H460 cells treated with vehicle control or 10 μM GSK343 for 72 h were analyzed for BRCA1 mRNA levels by qRT-PCR. The results (mean±SD) are expressed as relative mRNA levels compared to that obtained for the Control cells (assigned a value of 1)(left). Lysates were immunoblotted with the indicated antibodies (right). C. Soluble chromatin from H460 cells was precipitated with anti-EZH2 (left), anti-MUC1-C (right) or a control IgG. D. In the re-ChIP analysis, EZH2 precipitates were released and re-immunoprecipitated with anti-MUC1-C and a control IgG. E. Soluble chromatin from H460/CshRNA and H460/MUC1shRNA cells was precipitated with anti-H3K27me3 or a control IgG. The final DNA samples were amplified by qPCR with primers for the *BRCA1* promoter. The results (mean±SD of three determinations) are expressed as the relative fold enrichment compared with that obtained with the IgG control (assigned a value of 1).

Table S1. Primers used for qRT—PCR.

MUC1 qRT-F	AAAGCGATGGCGATTGGG
MUC1 qRT-R	CTCACCAGCCCAAACAGG
EZH2 qRT-F	ATTTCGTAGGAGGGAGCAAAG
EZH2 qRT-R	TGGGCCTGCTACTGTTATTG
SUZ12 qRT-F	GCAGCTTACGTTTACTGGTTTC
SUZ12 qRT-R	TGAGTTTGGTGATGGCTTATCT
EED qRT-F	TAAGGGCACGTAGAGCATTTAG
EED qRT-R	TGAGCAGGAAGACAGTACAAAG
NF-κB-p65 qRT-F	TGAGCCCACAAAGCCTTATC
NF-κB-p65 qRT-R	ACAATGCCAGTGCCATACA
E-cadherin qRT-F	GAACAGCACGTACACAGCCCT
E-cadherin qRT-R	GCAGAACTGTCCCTGTCCCAG
BRCA1 qRT-F	CCTTCTACTGTCCTGGCTACTA
BRCA1 qRT-R	CAGATTTCCAAGGGAGACTTCA
GAPDH qRT-F	CCATGGAGAAGGCTGGGG
GAPDH qRT-R	CAAAGTTGTCATGGATGACC

Table S2. Primers used for ChIP qPCR.

**ChIP-qPCR primers *EZH2* promoter**	
qF	AAAGCGATGGCGATTGGG
qR	TCCACTGCCTTCTGAGTCC
**ChIP-qPCR primers *EZH2* intron-1**	
qF	GCCTCAAGTCTCCTTTGTGT
qR	CCCACCAACTTGTGTCTGT
**ChIP-qPCR primers for *CDH1* promoter**	
qF	CTTTCTGATCCCAGGTCTTAGTG
qR	TAGGGTCTAGGTGGGTTATGG
**ChIP-qPCR primers for *BRCA1* promoter**	
qF	AAAGCGATGGCGATTGGG
qR	TCCACTGCCTTCTGAGTCC
**ChIP qPCR primer *GAPDH* promoter**	
qF	TACTAGCGGTTTTACGGGCG
qR	TCGAACAGGAGGAGCAGAGAGCGA

Figure 1C L

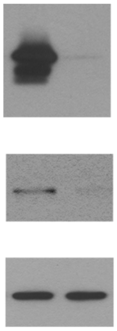

Figure 1C R

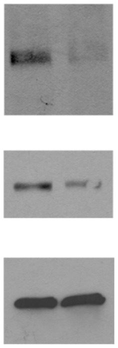

Figure 1D

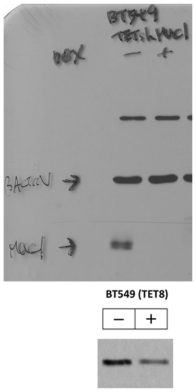

Figure 1E

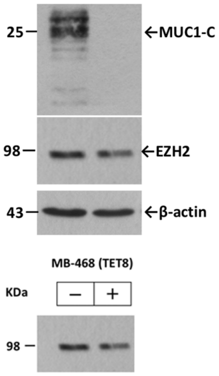

Figure 2B left

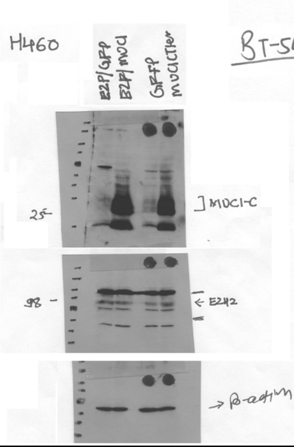

Figure 2B right

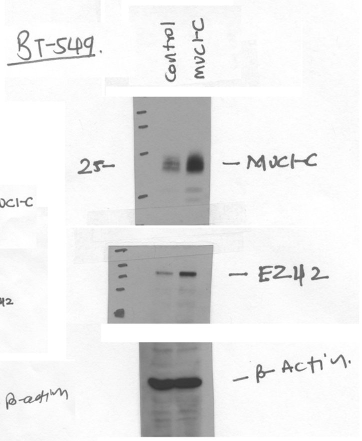

Figure 2D

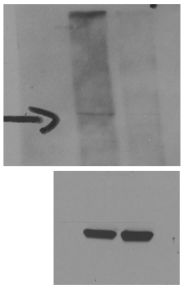

Figure 2E

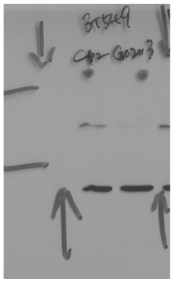

Figure 2F

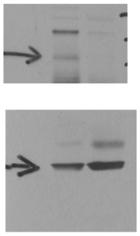

Figure 3D left
p-pRB

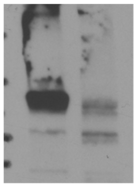

Figure 3D right
p-pRB

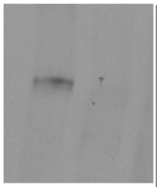

Figure 4C

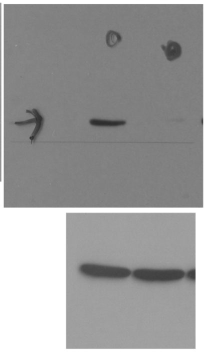

Figure 5D

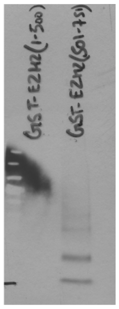

Figure 5E left

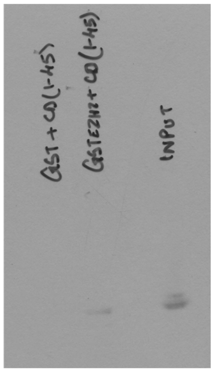

5E right

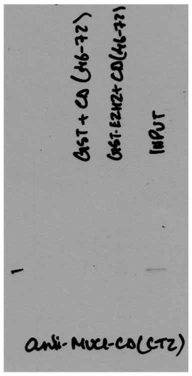

Figure 5F

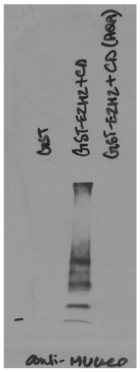

Figure 5G

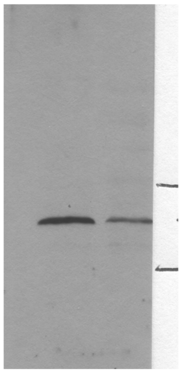

Figure 6A

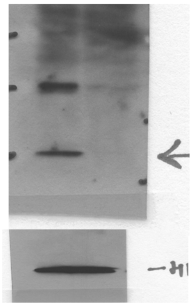

6B

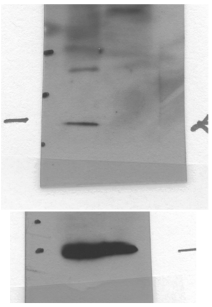

6C

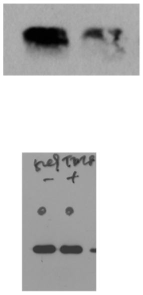

6D

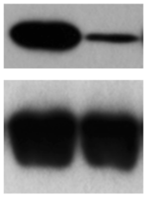

Figure 7A

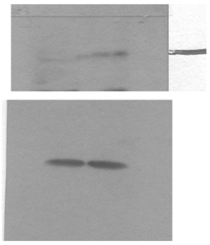

Figure 7 B

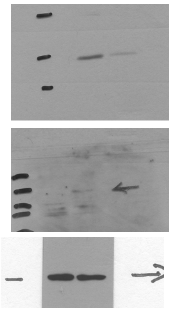

Supplement: Supplementary file 1 — Supplementary Material [file 41598_2017_7850_MOESM1_ESM.pdf]
